# Prostaglandin E2 and F2alpha Modulate Urinary Bladder Urothelium, Lamina Propria and Detrusor Contractility via the FP Receptor

**DOI:** 10.3389/fphys.2020.00705

**Published:** 2020-06-23

**Authors:** Zane Stromberga, Russ Chess-Williams, Christian Moro

**Affiliations:** Centre for Urology Research, Faculty of Health Sciences & Medicine, Bond University, Gold Coast, QLD, Australia

**Keywords:** inflammatory mediators, prostaglandins, prostaglandin antagonists, urinary bladder, overactive bladder, bladder inflammation, urothelium, mucosa

## Abstract

Current pharmacological treatment options for many bladder contractile dysfunctions are not suitable for all patients, thereby bringing interest to the investigation of therapies that target a combination of receptors. This study aimed to compare responses of PGE_2_ on the urinary bladder urothelium with lamina propria (U&LP, also called the bladder mucosa) or detrusor smooth muscle and attempt to identify the receptor subtypes mediating PGE_2_ contractile responses in these tissues. In the presence of selective EP1 – 4 receptor antagonists, varying concentrations of PGE_2_ were applied to isolated strips of porcine U&LP and detrusor that were mounted in organ baths filled with Krebs-bicarbonate solution and gassed with carbogen. The addition of PGE_2_ (1 and 10 μM) and PGF_2α_ (10 μM) to U&LP preparations caused significant increases in the baseline tension and in the spontaneous phasic contractile frequency. In detrusor preparations, significant increases in the baseline tension were observed in response to PGE_2_ (1 and 10 μM) and PGF_α_ (10 μM), and spontaneous phasic contractions were initiated in 83% of preparations. None of the selective PGE_2_ receptor antagonists inhibited the increases in baseline tension in both U&LP and detrusor. However, the antagonism of PGF_2α_ receptor showed significantly inhibited contractile responses in both layers of the bladder. This study presents prostaglandin receptor systems as a potential regulator of urinary bladder contractility. The main contractile effects of PGE_2_ in both U&LP and detrusor are mediated via the FP receptor with no observed contribution from any of the four EP receptors.

## Introduction

Bladder contractile dysfunctions, such as overactive bladder (OAB) and underactive bladder (UAB), are common lower urinary tract dysfunctions that lower the overall quality of life in people living with it (Haylen et al., 2010; D’ancona et al., 2019), heightens the likelihood of developing depression and anxiety, as well as increase healthcare usage (Milsom et al., 2012). Despite extensive research investigating the mechanisms that potentially mediate bladder contractions, the cause and development of many lower urinary tract dysfunctions are poorly understood. Bladder contractions are under the control of the parasympathetic nervous system (De Groat et al., 1981) and mediated by the release of acetylcholine that acts upon the M2 and M3 muscarinic receptors found in the detrusor smooth muscle (Hegde et al., 1997; Sigala et al., 2002). It has been recognized that the urothelium, which lines the lumen of the bladder, is capable of responding to mechanical, chemical and thermal stimuli (Birder, 2006; Stromberga et al., 2019, 2020b). Furthermore, it is capable of releasing several signaling molecules in response to stretch, including acetylcholine (Moro et al., 2011) ATP and nitric oxide (Ferguson et al., 1997; Fry and Vahabi, 2016) that are involved in the modulation of the micturition reflex. Myofibroblasts (also referred to as interstitial cells) found in the underlying lamina propria are thought to be involved in modulating bladder behavior by amplifying sensory responses to stretch occurring during the filling phase (Fry et al., 2007). Therefore, the involvement of the urothelium and the underlying lamina propria should be considered as potential contributors to bladder contractile pathology.

Prostaglandins have been suggested to be involved in the modulation of bladder function for decades. They are synthesized from arachidonic acid that is released from the cell membrane via the hydrolysis of the SN-2 bond by the phospholipase A2 enzyme (PLA2) (Clark et al., 1991). Prostaglandins can also be produced locally within the U&LP and smooth muscle layers in the human urinary bladder (Abrams et al., 1979; Jeremy et al., 1987). Two cyclooxygenase isoforms COX-1 and COX-2 metabolize arachidonic acid into PGH_2_, which is subsequently converted into five primary prostaglandins via their respective synthases: PGE_2_, PGD_2_, PGF_2α_, PGI_2_ and TXA_2_ (Woodward et al., 2011). These signaling molecules exert their function through the stimulation of nine specific G-protein coupled receptors (GPCRs): EP1–EP4, DP1, DP2, FP2, IP, and TP, respectively. Both COX-1 and COX-2 are expressed within the bladder wall (De Jongh, 2007, 2009) specifically on the urothelium, lamina propria and on the surface of the inner muscle bundles of the detrusor. COX-1 is predominately expressed in the basal and intermediate layers of the urothelium and on the interstitial cells located in the lamina propria, indicating that these cells are capable of respond to prostaglandins (De Jongh, 2009). The production of prostaglandins differs between species. For example, in the rat bladder, PGI_2_ is the major prostaglandin produced (Jeremy et al., 1984), whereas in the rabbit bladder it is PGE_2_ (Leslie et al., 1984). In the human bladder, the primary prostaglandins synthesized are PGI_2_, followed by PGE_2,_ PGF_2α_, and TXA_2_ (Jeremy et al., 1984; Masunaga et al., 2006). However, there are significant differences in the bladder contractile responses between species for the different prostaglandin receptor subtypes (Root et al., 2015).

Studies involving human detrusor have shown that the release of prostaglandins has direct influences on the micturition reflex (Bultitude et al., 1976; Andersson et al., 1977). Overproduction of prostaglandins have been observed in several conditions, including bladder outlet obstruction (Masick et al., 2001), bladder overactivity due to spinal cord injury (Masunaga et al., 2006), and in inflammation (Wheeler et al., 2001). Furthermore, contractions of the detrusor smooth muscle in response to acetylcholine and ATP are enhanced by prostaglandins in both rabbit (Anderson, 1982) and guinea-pig animal models (Nile et al., 2010). There are complex interactions between the three chemicals, as ATP enhances PGE_2_ release via a P2-purinoreceptor-mediated mechanism (Kasakov and Vlaskovska, 1985), and acetylcholine is a modulator of PGE_2_ release (Nile and Gillespie, 2012). The interactions between these mediators can potentially result in amplification of molecule signaling and therefore in increased contractile activity, further supporting the interest in investigating prostaglandin involvement in contractile disorders such as OAB. Other research has also shown interest in prostaglandin release from the lower urinary tract. For example, the presence of PGE_2_ in the urine has been linked to bladder dysfunction and proposed as a potential future biomarker (Reyes and Klahr, 1990). To further this, urine samples collected from female patients with overactive bladder showed that there were significant increases in the PGE_2_ and PGF_2α_ levels when compared with the control group (Kim et al., 2006).

Prostaglandin E_2_, in particular, is thought to be the most likely contributor to bladder disorders as it can modulate bladder function via afferent signaling (Andersson, 2010) and potentially detrusor overactivity by sensitizing capsaicin-sensitive afferent nerve endings (Maggi, 1988; Park et al., 1999). It is released upon degranulation of mast cells (Schmauder-Chock and Chock, 1989) together with histamine, which is also capable of impacting the bladder contractility (Stromberga et al., 2019, 2020a). Moreover, PGE_2_ is produced locally in the bladder in response to distension and inflammation (Funk, 2001). Notably, the excitatory effects exerted on the bladder wall via the stimulation of PGE_2_ receptors has previously been studied in the rat (Chuang et al., 2012) and mouse (Schroder et al., 2004; Mccafferty et al., 2008) animal models. There are four identified prostaglandin E_2_ receptor subtypes: EP1, EP2, EP3, and EP4. The presence of the EP1 and EP2 receptor subtypes has been shown in guinea pig urothelium and lamina propria (Rahnama’i et al., 2010) and the expression of all four PGE_2_ receptor subtypes (EP1–EP4) has been identified in the canine bladder using *in situ* hybridization and immunohistochemistry (Ponglowhapan et al., 2010). Generally, activation of EP1 and EP3 receptors is associated with an excitatory response, whereas EP2 and EP4 are associated with inhibition of smooth muscle contractility (Woodward et al., 2011). Furthermore, clinical uses of the prostaglandin E_2_ agonist dinoprostone, include the stimulation of uterine smooth muscle contractions during labor (Stephenson and Wing, 2015) with the prostaglandin F_2α_ agonist, carboprost, being a viable alternative to oxytocin during the management of labor (Sunil Kumar et al., 2016). Therefore, the involvement of EP1, EP3 and FP receptors is of particular interest in the modulation of lower urinary tract contractility due to their excitatory effect when stimulated.

Alterations in bladder physiology in disease states has been thought to primarily occur through the regulation of detrusor smooth muscle contractility. However, it is increasingly recognized that the bladder urothelium with lamina propria (U&LP, also called the bladder mucosa) plays an important role in the overall contractility of the bladder and in its intracellular signaling. As such, recent research has focused on the effects of pharmacological mediators on the physiology of the U&LP and the detrusor as separate layers. This study aimed to compare responses of the urothelium with lamina propria to PGE_2_ with those of the detrusor and identify the receptor subtypes responsible for the PGE_2_-mediated contractions in these tissues.

## Materials and Methods

### Tissue Preparation

Urinary bladders were obtained from female Large-White-Landrace pigs (approximately 6 months old, with a live-weight of 80–100 kg) from the local abattoir after slaughter for the routine commercial provision of food. Isolated strips (10 mm × 5 mm) of porcine bladder were removed from the anterior wall of the bladder dome region and urothelium with lamina propria (U&LP) carefully dissected from the underlying detrusor layer. Two adjacent strips were taken from each animal’s bladder and paired together as control-experimental tissues, with each paired strip preparation expressed as “*n*” where comparisons or changes are recorded. *n* equates to the number of individual bladders used in this study.

Strips of U&LP and detrusor tissue were suspended in 10 mL organ baths (Labglass, Brisbane, QLD, Australia) containing Krebs-bicarbonate solution (NaCl 118.4 mM, NaHCO_3_ 24.9 mM, CaCl_2_ 1.9 mM, MgSO_4_ 2.41 mM, KCl 4.6 mM, KH_2_PO_4_ 1.18 mM, and D-glucose 11.7 mM) at 37°C and perfused with a gas mixture of 95% oxygen and 5% carbon dioxide. The preparations were washed three times with Krebs-bicarbonate solution and tension adjusted to approximately 2 g, which then became the tissue baseline. A single dose of agonist was added to the U&LP and detrusor preparations after an incubation period with an antagonist or vehicle control. The concentrations specific for each receptor subtype were determined using affinity values appearing in the literature utilizing similar methodology and isolated tissue bath experiments. Tissue strips exposed to antagonists or vehicle controls were incubated for 30 min to allow full equilibration with the receptor. Each experiment was completed within 20 min after the addition of the agonist. Baseline tension, frequency and amplitude of spontaneous phasic contractions were recorded simultaneously using isometric force transducers (MCT050/D, ADInstruments, Castle Hill, NSW, Australia) on a PowerLab system using LabChart v7 software (ADInstruments). Porcine urinary bladders are a well-recognized animal model for bladder research (Cheng et al., 2011; Parsons et al., 2012; Mitsui et al., 2019; Stromberga et al., 2019) with similar physiology and pharmacology to human bladders. As no animals were bred, harmed, culled, interfered, or interacted with as part of this research project, Animal Ethics Approval was not required for bladder use (Queensland Government, 2016).

### Pharmaceutical Agents

Prostaglandin E_2_, F_2__α_, EP1 receptor antagonist SC-19220, EP1, and EP2 receptor antagonist AH6809, EP3 receptor antagonist L-798106, EP4 receptor antagonist AH23848, FP receptor antagonist AL-8810 and ATP receptor agonist α,β-methylene ATP were obtained from Cayman Chemicals, Ann Arbor, MI, United States. The muscarinic receptor antagonist atropine, cyclooxygenase (COX) inhibitor indomethacin, and the nitric oxide synthase inhibitor N_ω_-Nitro-L-arginine were obtained from Sigma Aldrich, St. Louis, MO, United States. Prostaglandin E_2_, prostaglandin F_2α_, AH6809 and indomethacin were dissolved in 100% ethanol and diluted as needed with distilled H_2_O. Atropine, N_ω_-Nitro-L-arginine, and α,β-methylene ATP were dissolved in distilled H_2_O and diluted as required. SC19220, L-798196, AH23848, and AL-8810 were dissolved in DMSO and diluted with distilled H_2_O.

### Experimental Procedure

A single dose of prostaglandin E_2_ (1 or 10 μM) or prostaglandin F_2α_ (10 μM) was applied to an isolated strip of U&LP or detrusor after a 30-minute equilibration period with a selective receptor antagonist. Baseline tensions and the amplitude and frequency of spontaneous phasic contractions of U&LP were measured before the application of the agonist and 5 min after addition. As spontaneous contractions were only observed in 21% of detrusor samples, only baseline tension was measured (before the application of the agonist, 10 min after and 20 min after).

### Measurements and Statistical Analysis

The changes in the baseline tension were measured before the addition of an agonist and at the peak contractile response. The frequency of spontaneous contractions was measured over a period of 5 min and expressed as the number of spontaneous phasic contraction cycles per minute (cpm). The average amplitude was measured from base to peak of each spontaneous contraction. Measurements for frequency and amplitude were taken before any agonist was added and during the peak contractile response to the agonist. Data was graphed and analyzed using GraphPad Prism version 8.1.1 for Windows (GraphPad Software, La Jolla, CA, United States) and results shown as the mean change ± SEM. Responses between control and experimental tissues were compared using a Student’s *t*-test with *p* < 0.05 considered significant.

## Results

### Prostaglandin E_2_ on U&LP Preparations

**FIGURE 1:**
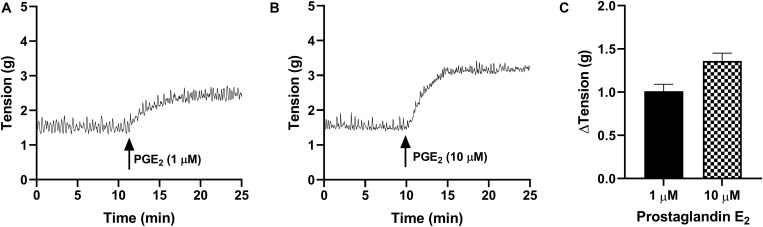
Traces showing changes to baseline tension of U&LP in response to PGE_2_ at concentrations of 1 μM **(A)** and 10 μM **(B)** over 15 min. **(C)** Peak responses after the addition of PGE_2_ (1 μM, *n* = 38) and PGE_2_ (10 μM, *n* = 42).

In the absence of prostaglandin E_2_ or any receptor antagonists, strips of urothelium with lamina propria (U&LP) exhibited spontaneous phasic contractions at frequencies of 3.34 ± 0.06 cycles per min^–1^ (cpm) with amplitudes of 0.52 ± 0.02 g (*n* = 215 for both). In response to prostaglandin E_2_ (1 μM), U&LP baseline tensions increased by 91 ± 9% (*n* = 38, *p* < 0.001), the frequency of spontaneous phasic contractions increased by 39 ± 7% (*n* = 38, *p* < 0.001) and amplitudes decreased by 18 ± 5% (*n* = 38, *p* < 0.001) during the peak response (Table 1 and Figure 1). The addition of an even higher concentration of prostaglandin E_2_ (10 μM) caused increases in the U&LP baseline tensions by 106 ± 9% (*n* = 42, *p* < 0.001), frequencies of spontaneous phasic contractions by 40 ± 10% (*n* = 42, *p* < 0.01) and amplitude decreases by 28 ± 6% (*n* = 22, *p* < 0.001) during peak response (Table 1 and Figure 1).

**TABLE 1:** U&LP effect of prostaglandin E_2_ (1 and 10 μM) on the baseline tension and on the frequency and amplitude of spontaneous phasic contractions (mean change ± SEM).

PGE_2_ conc.	ΔTension (g)	ΔFrequency (cpm)	ΔAmplitude (g)	*n*
1 μM	1.01 ± 0.08***	1.13 ± 0.19***	−0.14 ± 0.04***	38
10 μM	1.36 ± 0.09***	1.19 ± 0.30**	−0.16 ± 0.03**	42

**p < 0.05, ***p < 0.001. Paired Student’s two-tailed *t*-test.

### Prostaglandin E_2_ on Detrusor Preparations

**FIGURE 2:**
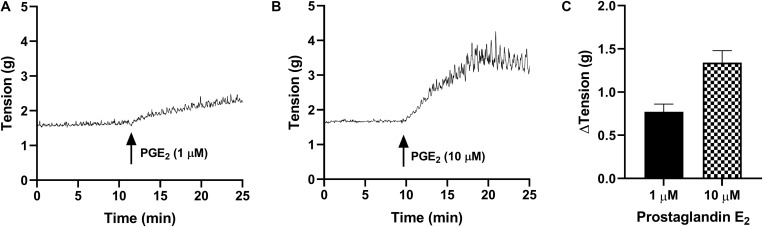
Traces showing changes to baseline tension of detrusor smooth muscle in response to PGE_2_ at concentrations of 1 μM **(A)** and 10 μM **(B)** over 15 min. **(C)** Peak responses after the addition of PGE_2_ (1 μM, *n* = 38) and PGE_2_ (10 μM, *n* = 34).

In detrusor preparations, PGE_2_ caused concentration-dependent increases in baseline tension over the increasing concentrations (1–10 μM, Table 2 and Figure 2). Detrusor preparations did not reach a visible peak in response to PGE_2_ within the experimental timeframe, so each tension recording was completed at set intervals of 10 and 20 min. In response to prostaglandin E_2_ (1 μM, Table 2 and Figure 2), an increase of 80 ± 13% (*n* = 38, *p* < 0.001) was observed 10 min after the addition of the agonist, and in response to prostaglandin E_2_ (10 μM, Table 2 and Figure 2) an increase of 155 ± 21% (*n* = 34, *p* < 0.001) was observed 10 min after the addition of agonist. Twenty minutes after the addition of prostaglandin E_2_ (1 μM, Table 2), baseline tensions had increased by 107 ± 13% (*n* = 38, *p* < 0.001), whereas 20 min after the treatment with prostaglandin E_2_ (10 μM, Table 2), had increased by 175 ± 21% (*n* = 34, *p* < 0.001).

**TABLE 2:** Detrusor effect of prostaglandin E_2_ (1 and 10 μM) on the baseline tension and on the frequency and amplitude of spontaneous phasic contractions (mean change ± SEM).

PGE_2_ conc.	ΔTension (g) at 10 min	ΔTension (g) at 20 min	*n*
1 μM	0.77 ± 0.09***	1.06 ± 0.09***	38
10 μM	1.34 ± 0.14***	1.46 ± 0.11***	34

***p < 0.001. Paired Student’s two-tailed *t*-test.

Only 21% of all detrusor preparations exhibited spontaneous phasic contractions at baseline and as such, the frequency and amplitude of spontaneous activity was not calculated. Of those that did not exhibit baseline activity, spontaneous phasic contractions were initiated after the addition of PGE_2_ in 83% of preparations.

### Receptor-Selective Antagonists in U&LP Preparations

The responses to prostaglandin E_2_ were observed in the presence of the selective receptor antagonists: SC-19220 (EP1 antagonist, 10 μM), AH6809 (EP1/EP2 antagonist, 10 μM), L-798106 (EP3 antagonist, 100 nM) and AH23848 (EP4 antagonist, 10 μM). None of the prostaglandin E_2_ receptor antagonists had any effect on baseline tension, frequency or amplitude of spontaneous phasic contractions and they also had no effect on U&LP in response to prostaglandin E_2_ (1 μM, Table 3). The time required to reach peak baseline tension was investigated in U&LP tissues treated with PGE_2_ in the presence and absence of EP receptor antagonists. In response to prostaglandin E_2_ (1 μM), U&LP tissue strips took 4.48 ± 0.28 min to reach their peak baseline tensions. Tissues incubated with SC-19220 (1 μM, *n* = 8, *p* < 0.05) took 6.37 ± 0.39 min to reach peak. There were no significant differences in times taken to reach peak baseline tension in tissues treated with either AH6809, L-798106 or AH23848.

**TABLE 3:** Baseline tension and phasic contraction responses to U&LP treatment with prostaglandin E_2_ (1 μM) in the absence and presence of prostaglandin E_2_ receptor antagonists (mean change ± SEM).

		ΔTension (g)		ΔFrequency (cpm)		
			
Antagonist	Conc.	Absence	Presence	Absence	Presence	*n*
SC-19220	10 μM	1.01 ± 0.19	1.27 ± 0.24	1.32 ± 0.43	1.88 ± 0.38	8
AH6809	10 μM	1.12 ± 0.19	1.14 ± 0.20	1.42 ± 0.53	1.23 ± 0.41	8
L-798106	100 nM	0.97 ± 0.09	0.82 ± 0.10	0.68 ± 0.15	0.80 ± 0.18	8
AH23848	10 μM	0.90 ± 0.10	0.99 ± 0.13	1.05 ± 0.36	1.61 ± 0.44	12

*None of the antagonists had statistically significant effects using a paired Student’s two-tailed *t*-test.*

**FIGURE 3:**
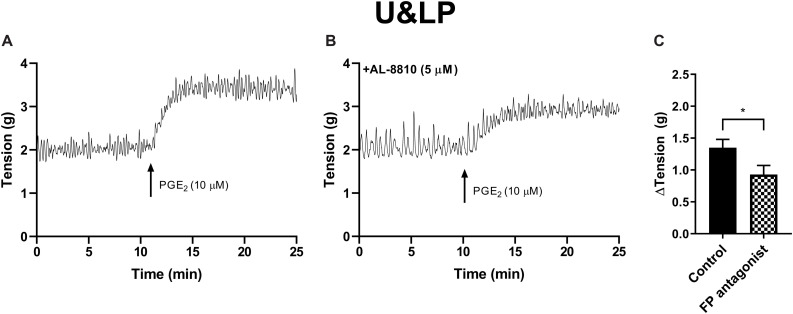
U&LP responses to PGE_2_ (10 μM) **(A)** and in the presence of FP receptor antagonist AL-8810 (5 μM) **(B)**. Increases in the baseline tension in response to PGE_2_ are represented as mean change ± SEM **(*C*)**. Changes in the baseline tension between control and experimental conditions were evaluated using a paired Student’s two tailed *t*-test, where *p < 0.05.

The potential influence of receptor systems, other than prostaglandin E_2_, to contribute contractile responses to PGE_2_ were investigated. Neither the baseline tension, frequency nor amplitude of spontaneous phasic contractions in response to prostaglandin E_2_ (10 μM) were affected by the presence of the muscarinic receptor antagonist atropine (1 μM), cyclooxygenase (COX) inhibitor indomethacin (5 μM), nitric oxide synthase inhibitor N_ω_-Nitro-L-arginine (L-NNA, 100 μM) or P2X receptor desensitizing agonist α,β-methylene ATP (αβm-ATP, 10 μM, Table 4). The tissue did not exhibit changes in the tension or in the frequency of phasic contractions in response to the addition of the FP receptor antagonist AL-8810 (5 μM). However, the presence of AL-8810 (5 μM) significantly inhibited increases in the baseline tension in response to PGE_2_ (10 μM, *n* = 8, *p* < 0.05, Figure 3). In the absence of any antagonist, contractions to PGE_2_ increased the tension by 103 ± 12%. In the presence of AL-8810 (5 μM) this increase was inhibited with the remaining contraction being 77 ± 8% (*n* = 8, *p* < 0.05, Table 4).

**TABLE 4:** U&LP responses to prostaglandin E_2_ (10 μM) in the absence and presence of atropine (muscarinic antagonist), indomethacin (cyclooxygenase antagonist), N_ω_-Nitro-L-arginine (L-NNA, nitric oxide antagonist), α,β-methylene ATP (αβm-ATP, ATP agonist), and AL-8810 (FP antagonist) (mean change ± SEM).

		ΔTension (g)		ΔFrequency (cpm)		
			
Antagonist	Conc.	Absence	Presence	Absence	Presence	*n*
Atropine	1 μM	1.45 ± 0.18	1.55 ± 0.12	0.53 ± 0.18	0.86 ± 0.21	6
Indomethacin	5 μM	1.90 ± 0.25	1.79 ± 0.21	0.73 ± 1.29	2.11 ± 1.45	4
L-NNA	100 μM	2.00 ± 0.32	2.31 ± 0.42	2.86 ± 1.32	3.51 ± 1.78	4
αβm-ATP	10 μM	1.58 ± 0.35	1.99 ± 0.48	0.46 ± 0.22	0.49 ± 0.23	4
AL-8810	5 μM	1.35 ± 0.13	0.93 ± 0.14*	1.12 ± 0.38	0.68 ± 0.27	8

*p < 0.05. Paired Student’s two-tailed *t*-test.

### Receptor-Selective Antagonists in Detrusor Preparations

Detrusor preparations did not reach a visible peak in response to PGE_2_ (1 μM) within the experimental timeframe, so each tension recording was completed at set intervals of 10 and 20 min. As such, changes in detrusor tension were measured at 10 and 20 min after the addition of each agonist (Table 5). The responses to prostaglandin E_2_ were observed in the presence of the selective prostaglandin E_2_ receptor antagonists: SC-19220 (EP1 antagonist, 10 μM), AH6809 (EP1/EP2 antagonist, 10 μM), L-798106 (EP3 antagonist, 100 nM) and AH23848 (EP4 antagonist, 10 μM). None of the prostaglandin E_2_ receptor antagonists had any effect on baseline tension in detrusor tissue strips in response to prostaglandin E_2_ (1 μM).

**TABLE 5:** Detrusor responses to prostaglandin E_2_ (1 μM) at 10 and 20 min after the addition of agonist in the absence and presence of PGE_2_ receptor antagonists (mean change ± SEM).

		ΔTension (g) at 10 min		ΔTension (g) at 20 min		
			
Antagonist	Conc.	Absence	Presence	Absence	Presence	*n*
SC-19220	10 μM	0.60 ± 0.08	0.49 ± 0.11	1.12 ± 0.18	1.09 ± 0.15	8
AH6809	10 μM	0.64 ± 0.13	0.62 ± 0.10	0.96 ± 0.16	0.95 ± 0.16	8
L-798106	100 nM	1.12 ± 0.20	1.05 ± 1.16	1.25 ± 0.20	1.35 ± 0.26	10
AH23848	10 μM	0.46 ± 0.10	0.49 ± 0.14	0.81 ± 0.16	0.80 ± 0.16	8

*None of the antagonists had statistically significant effects (paired Student’s two-tailed *t*-test).*

**FIGURE 4:**
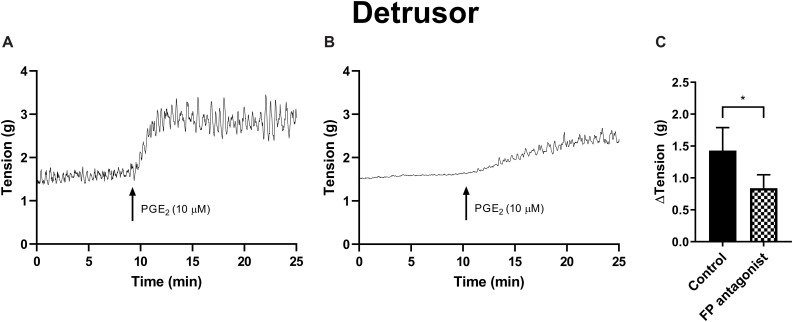
Detrusor responses to PGE_2_ (10 μM) **(A)** and in the presence of FP receptor antagonist AL-8810 (5 μM) **(B)**. Increases in the baseline tension in response to PGE_2_ are represented as mean change ± SEM **(C)**. Changes in the baseline tension between control and experimental conditions were evaluated using a paired Student’s two tailed *t*-test, where *p < 0.05.

The potential for receptor systems, other than prostaglandin E_2_, to contribute contractile responses to PGE_2_ were investigated. It was found that the baseline tension in detrusor preparations, in response to prostaglandin E_2_ (10 μM), was not affected by the presence of the muscarinic receptor antagonist atropine (1 μM), cyclooxygenase (COX) inhibitor indomethacin (5 μM), nitric oxide synthase inhibitor N_ω_-Nitro-L-arginine (L-NNA, 100 μM) or P2X receptor desensitizing agonist α,β-methylene ATP (αβm-ATP, 10 μM, Table 6). Treatment with the FP receptor antagonist AL-8810 (5 μM) did not affect baseline responses. In the presence of AL-8810 increases to baseline tension mediated by prostaglandin E_2_ (10 μM) were significantly inhibited both 10 and 20 min after the addition of the agonist (*n* = 8, *p* < 0.05 for both, Figure 4). In the absence of any antagonists, contractions to PGE2 increased the tension by 233 ± 60% at 10 min. In the presence of AL-8810 (5 μM) this increase was inhibited with the remaining contraction of 127 ± 46% (*n* = 8, *p* < 0.05, Table 6).

**TABLE 6:** Detrusor responses to prostaglandin E_2_ (10 μM) in the absence and presence of atropine (muscarinic antagonist), indomethacin (cyclooxygenase antagonist), N_ω_-Nitro-L-arginine (L-NNA, nitric oxide antagonist), α,β-methylene ATP (αβm-ATP, ATP agonist), and AL-8810 (FP antagonist) (mean change ± SEM).

		ΔTension (g) at 10 min		ΔTension (g) at 20 min		
			
Antagonist	Conc.	Absence	Presence	Absence	Presence	*n*
Atropine	1 μM	1.38 ± 0.21	1.02 ± 0.09	1.72 ± 0.11	1.47 ± 0.11	6
Indomethacin	5 μM	1.55 ± 0.55	1.29 ± 0.42	1.75 ± 0.55	1.35 ± 0.43	4
L-NNA	100 μM	2.21 ± 0.28	1.90 ± 0.32	2.05 ± 0.26	1.94 ± 0.27	4
αβm-ATP	10 μM	1.40 ± 0.21	1.72 ± 0.49	1.45 ± 0.29	1.94 ± 0.43	4
AL-8810	5 μM	1.43 ± 0.36	0.84 ± 0.21*	1.39 ± 0.22	1.01 ± 0.18*	8

*p < 0.05. Paired Student’s two-tailed *t*-test.

### Prostaglandin F_2α_ on U&LP and Detrusor Preparations

**FIGURE 5:**
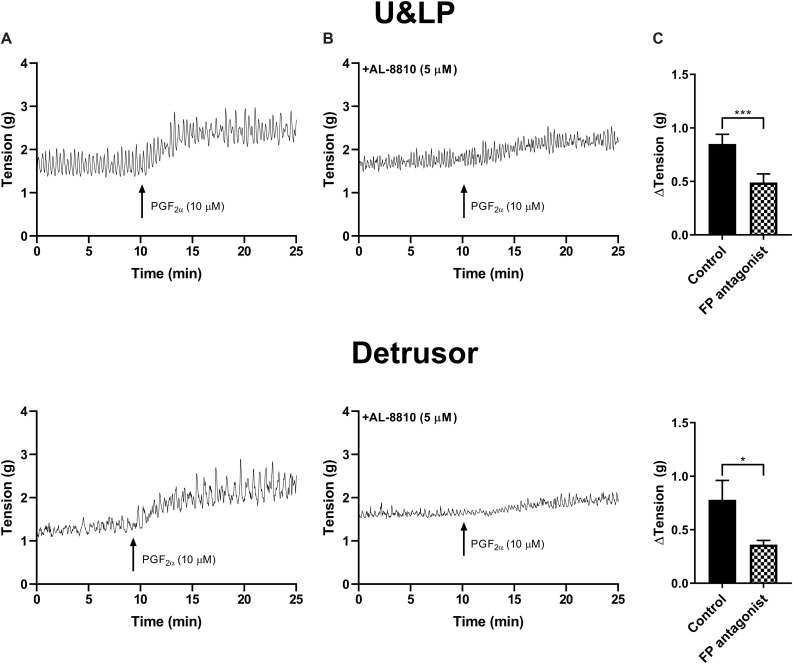
U&LP and detrusor responses to PGF_2α_ (10 μM) **(A)** and in the presence of FP receptor antagonist AL-8810 (5 μM) **(B)**. Increases in the baseline tension in response to PGF_2α_ are represented as mean change ± SEM **(C)**. Changes in the baseline tension between control and experimental conditions were evaluated using a paired Student’s two tailed *t*-test, where *p < 0.05, ***p < 0.001.

In preparations of U&LP, treatment with PGF_2α_ (10 μM) caused a significant increase in the baseline tension of 65 ± 9% (*n* = 14, *p* < 0.001). Additionally, the frequency of spontaneous phasic contractions increased by 13 ± 5% (*n* = 14, *p* < 0.05) with no changes observed in the amplitude of these contractions. In the presence of AL-8810 (FP antagonist, 5 μM), increases in the baseline tension in response to PGF_2α_ (10 μM) were significantly inhibited (*n* = 8, *p* < 0.001, Table 7 and Figure 5). However, the antagonism of FP receptor did not affect the frequency or amplitude of spontaneous phasic contractions.

**TABLE 7:** U&LP responses to PGF_2α_ (10 μM) in the absence and presence of AL-8810 on the baseline tension and on the frequency and amplitude of phasic contractions (top).

			PGF_2α_ with	
		PGF_2α_	AL-8810	*n*
Urothelium with lamina propria (U&LP)	ΔTension (g)	0.850.09	0.490.08***	8
	ΔAmplitude (g)	−0.110.06	−0.130.07	8
	ΔFrequency (cpm)	0.670.23	0.730.14	8
Detrusor	ΔTension (g) at 10 min	0.780.18	0.360.04*	8
	ΔTension (g) at 20 min	0.910.19	0.570.08	8

Detrusor baseline tension responses at 10 and 20 min to PGF_2__α_ (10 μM) in the absence and presence of AL-8810 (bottom). Results represented as mean change ± SEM. *p < 0.05, ***p < 0.001. Paired Student’s two-tailed *t*-test.

In detrusor preparations, an increase of 88 ± 10% (*n* = 12, *p* < 0.001) was observed in response to PGF_2α_ (10 μM). The presence of FP antagonist AL-8810 (5 μM) significantly inhibited increases in the baseline tension (*n* = 8, *p* < 0.05). The inhibition was maintained both 10 and 20 min after the addition of PGF_2α_ (10 μM, Table 7).

## Discussion

This study presents prostaglandin E_2_ as a potential contributor to the overall urinary bladder contractility and showcases the ability of isolated urothelium with lamina propria (also called the bladder mucosa) to respond to this inflammatory mediator. Prostaglandin E_2_ causes a baseline tension increase in both U&LP and detrusor tissue, as well as influences the frequency and amplitude of U&LP spontaneous phasic contractions. In addition, this study is the first to identify responses to PGE_2_ in the urothelium with lamina propria when separated from its underlying detrusor layer. The two layers are distinct in both structure and function, with the U&LP comprized of several layers of epithelial cells and an underlying connective tissue layer with muscularis mucosae, while the detrusor is composed primarily of smooth muscle cells. The U&LP not only provides a physical barrier between urine and the bladder wall but is also capable of releasing chemicals like acetylcholine which can influence the contractility of the detrusor (Moro et al., 2011). Furthermore, it is also capable of responding to external signals like noradrenaline (Moro et al., 2013), nitric oxide (Moro et al., 2012), the five primary prostaglandins (Stromberga et al., 2020b), and histamine (Stromberga et al., 2019, 2020a), as well as release ATP (Andersson et al., 2018). It is known that cells found in U&LP can express both EP1 and EP2 receptors (Rahnama’i et al., 2010) indicating that they are capable of responding to PGE_2_ and eliciting a contractile response. However, the exact mechanisms and the involvement of the different PGE_2_ receptor subtypes in the contractile responses observed are still unclear.

In this study, increases in the baseline tension in both U&LP and detrusor tissues that were treated with two concentrations of PGE_2_ (1–10 μM) were observed. The presence of PGE_2_ increased the frequency of spontaneous contractile activity, which was similar to responses after muscarinic receptor activation (Moro et al., 2011), the target of many first-line pharmaceuticals to treat overactive bladder. In detrusor preparations, which generally do not exhibit spontaneous phasic contractions, these contractions were initiated in most preparations in response to the addition of PGE_2_. Previous studies have shown that contractions in detrusor smooth muscle in response to acetylcholine and ATP are enhanced by the presence of prostaglandins in both rabbit (Anderson, 1982) and guinea-pig animal models (Nile and Gillespie, 2012). There are complex interactions between these three molecules, as ATP enhances PGE_2_ release via a P2-purinoreceptor-mediated mechanism (Kasakov and Vlaskovska, 1985) and acetylcholine release has been detected when unstretched preparations were exposed to PGE_2_ (Nile and Gillespie, 2012). Thereby, interactions between these three mediators can potentially result in the amplification of molecule signaling and an increase in contractile activity. However, the involvement of acetylcholine, ATP and nitric oxide in mediating the increases in both the baseline tension and the frequency of spontaneous phasic contractions were ruled out as the contractile responses were the same when respective receptors were selectively antagonized or desensitized. Therefore, even though previous research has shown that acetylcholine is released when tissue is exposed to PGE_2_ (Nile and Gillespie, 2012), the inhibition of muscarinic receptors revealed no changes in the magnitude of contractions in response to PGE_2_. Furthermore, treatment with indomethacin, which inhibits prostaglandin production via cyclooxygenase (COX), did not affect contractile responses.

In order to isolate the role of a specific PGE_2_ receptor subtype in mediating these changes to baseline tension and the frequency of spontaneous phasic contractions, selective receptor antagonists for EP1–EP4 receptors were used. The antagonists and concentrations selected for this study were based on prior research investigating smooth muscle contractility in rat prostate gland (Tokanovic et al., 2010). When U&LP and detrusor smooth muscle tissues were treated with each individual PGE_2_ receptor subtype antagonists (EP1–EP4), there were no differences observed in the contractile responses to PGE_2_ when compared to tissues with no antagonist treatment. Activation of EP1 and EP3 receptor subtypes mediate contractions in smooth muscle, whereas EP2 and EP4 are involved in the relaxatory responses (Woodward et al., 2011). In this study, when both U&LP and detrusor tissues were treated with the highly selective EP1 receptor antagonist SC19220 with a pA_2_ value of approximately 5.9 (Jones et al., 2009) and EP3 receptor antagonist L798106 with a K*i* of 0.3 nM (Juteau et al., 2001) there was no change in the magnitude of contractions to PGE_2_. However, when U&LP was incubated with the EP1-selective receptor antagonist SC-19220, it took significantly more time to reach peak contraction in response to PGE_2_ compared to control tissues. Even though the stimulation of EP2 receptors has previously been shown to relax non-vascular urogenital smooth muscle tissue (Coleman et al., 1994; Tokanovic et al., 2010), no changes in contractile response were observed in tissue preparations that were treated with the dual EP1 and EP2 antagonist AH6809 [pA for EP1 approximately 7.3 and pA_2_ for EP2 approximately 5.7 (Jones et al., 2009)]. The involvement of EP4 receptors in the inhibition of muscle contractions was also ruled out using AH23848, an antagonist for EP4 receptors (pA_2_ value 5.4) with no activity on other EP receptor subtypes (Coleman et al., 1994). The lack of responses also aligns with previous findings suggesting that the EP4 receptor is involved in vascular smooth muscle relaxation instead of smooth muscle of the urinary bladder (Coleman et al., 1994; Tokanovic et al., 2010). Findings of this study suggest that the contractile responses observed to prostaglandin E_2_ in both U&LP and detrusor may be mediated by receptors systems other than EP, purinergic and cholinergic.

The ability of AL-8810 to significantly inhibit the contractile response to PGE_2_ suggests a mechanism of action at the FP receptor. This is most likely a result of the PGE_2_ chemical undergoing a conversion from PGE_2_ into PGF_2α_ through 9-ketoreductase activity upon contact with the tissue. The potential for these conversions has been known for some time (Canete Soler et al., 1987; Cheung and Challis, 1989; Abadir and Siragy, 2015). There is evidence to support a partial agonist effect of AL-8810 (Griffin et al., 1999). Although the addition of this antagonist showed no response, or any effect on the baseline activity. The contraction remaining after FP receptor inhibition is unknown, although does not appear to be related to stimulation of the muscarinic, purinergic or nitric oxide receptor systems, nor from the creation of new prostaglandins. One potential mechanism of action could be via a receptor unspecific pathway, such as affecting calcium channels, and this could be considered for future experiments.

A limitation of this study was the use of single-dose applications of PGE_2_ and PGF_2α_ to examine changes in frequencies and amplitudes of phasic contractions over a 20-minute timeframe. It would be of interest to examine a larger range of concentrations, as well as incorporate alternative agonists to obtain a wider understanding of the prostaglandin responses. Finally, there remains no literature showing FP receptor expression or localization in porcine or any other animal model bladders, and its identification throughout this region would assist in a better understanding of this receptor system. It should also be considered that PGE_2_ exhibits a similar affinity to PGF_2α_ on the FP receptor (Abramovitz et al., 2000), and as such, there is the potential for an influence of FP receptors in the responses observed. This cross-sensitivity of FP receptors to PGE_2_ may also present a mechanism underlying the inhibitory effect observed from treatment with AL-8810 and should be further explored in follow-up studies. Future studies could also investigate whether the suppressed responses observed after AL-8810 treatment is due to the actions of PGE_2_ after a possible conversion to PGF_2α_.

This study identifies a functional response in both U&LP and detrusor preparations to PGE_2_, and its ability to be inhibited by an FP receptor antagonist suggests some conversion to PGF_2α_ upon contact with the tissue. The response of direct application of PGF_2α_ which appears to be similar to the PGE_2_ response also supports this hypothesis. Thereby, the finding of this study supports and further advances prior research, where PGE_2_ and PGF_2α_ had previously been found to contract isolated detrusor smooth muscle in human preparations (Abrams and Feneley, 1975; Andersson et al., 1977; Palea, 1998).

## Conclusion

Prostaglandin E_2_ and prostaglandin F_2α_ can elicit clear contractile responses in both urinary bladder urothelium with lamina propria, and detrusor smooth muscle preparations. Furthermore, the addition of these prostaglandins in U&LP preparations increases the frequency of spontaneous phasic contractions. In the detrusor, which normally does not exhibit spontaneous contractions, these contractions were initiated in most preparations. The antagonism of all four PGE_2_ receptor subtypes, cholinergic receptors and purinergic receptors failed to inhibit contractile responses observed to the prostaglandins in either U&LP or detrusor tissues. However, the inhibition of EP1 receptor in U&LP resulted in a significant decrease in the time taken to reach peak contractile responses to PGE_2_ suggesting partial mediation via the EP1 receptor system. Antagonism of the FP receptor inhibited responses to PGF_2α_, but also to PGE_2_, suggesting some conversion of this into PGF_2α_ after contact with the tissue. Overall, contractile responses to both prostaglandin E_2_ and prostaglandin F_2α_ appear mediated, in part, by the FP receptor in both U&LP and detrusor smooth muscle.

## Data Availability Statement

The datasets generated for this study are available on request to the corresponding author.

## Ethics Statement

Ethical review and approval was not required for the animal study because bladders were obtained from the local commercial abattoir after slaughter for the routine commercial provision of food. As no animals were bred, harmed, culled, interfered, or interacted with as part of this research project, Animal Ethics Approval was not required for bladder use (reference cited in section “Tissue Preparation”).

## Author Contributions

ZS collected the data. All authors were all equally responsible for the study design, data analysis, preparation of manuscript, approved the final manuscript, and are accountable for all aspects of the work. All persons designated as authors qualify for authorship, and all those who qualify for authorship are listed.

## Conflict of Interest

The authors declare that the research was conducted in the absence of any commercial or financial relationships that could be construed as a potential conflict of interest.

## Abbreviations

ATP: adenosine triphosphate
OAB: overactive bladder
PGE_2_: prostaglandin E_2_
PGF_2 α_: prostaglandin F_2 α_
SEM: standard error of the mean
U&LP: lamina propria with urothelium
UAB: underactive bladder
αβ m-ATP: α β− methylene ATP.
